# Polyphenols as Inhibitors of Antibiotic Resistant Bacteria—Mechanisms Underlying Rutin Interference with Bacterial Virulence

**DOI:** 10.3390/ph15030385

**Published:** 2022-03-21

**Authors:** Marija Ivanov, Katarina Novović, Milka Malešević, Miroslav Dinić, Dejan Stojković, Branko Jovčić, Marina Soković

**Affiliations:** 1 Institute for Biological Research “Siniša Stanković”—National Institute of Republic of Serbia, University of Belgrade, Bulevar despota Stefana 142, 11000 Belgrade, Serbia; dejanbio@ibiss.bg.ac.rs (D.S.); mris@ibiss.bg.ac.rs (M.S.); 2 Institute of Molecular Genetics and Genetic Engineering, University of Belgrade, Vojvode Stepe 444A, 11042 Belgrade, Serbia; katarinanovovic@imgge.bg.ac.rs (K.N.); milkam@imgge.bg.ac.rs (M.M.); mdinic@imgge.bg.ac.rs (M.D.); bjovcic@bio.bg.ac.rs (B.J.); 3 Faculty of Biology, University of Belgrade, Studentski Trg 16, 11000 Belgrade, Serbia

**Keywords:** polyphenols, rutin, antibiotic resistance, bacteria, antimicrobial activity, antibiofilm activity, cytotoxicity, virulence, mechanism of activity

## Abstract

The rising incidence of antibiotic resistant microorganisms urges novel antimicrobials development with polyphenols as appealing potential therapeutics. We aimed to reveal the most promising polyphenols among hesperetin, hesperidin, naringenin, naringin, taxifolin, rutin, isoquercitrin, morin, chlorogenic acid, ferulic acid, *p*-coumaric acid, and gallic acid based on antimicrobial capacity, antibiofilm potential, and lack of cytotoxicity towards HaCaT, and to further test its antivirulence mechanisms. Although the majority of studied polyphenols were able to inhibit bacterial growth and biofilm formation, the most promising activities were observed for rutin. Further investigation proved rutin’s ability to prevent/eradicate *Pseudomonas aeruginosa* and MRSA urinary catheter biofilms. Besides reduction of biofilm biomass, rutin antibiofilm mechanisms included reduction of cell viability, exopolysaccharide, and extracellular DNA levels. Moderate reduction of bacterial adhesion to human keratinocytes upon treatment was observed. Rutin antivirulence mechanisms included an impact on *P. aeruginosa* protease, pyocyanin, rhamnolipid, and elastase production and the downregulation of the *lasI*, *lasR*, *rhlI*, *rhlR*, *pqsA* and *mvfR* genes. Rutin also interfered with membrane permeability. Polyphenols could repress antibiotic resistant bacteria. Rutin has shown wide antimicrobial and antibiofilm capacity employing a range of mechanisms that might be used for the development of novel antimicrobials.

## 1. Introduction

The current climate of antimicrobial resistance (AMR) is leading to a potential global health crisis. Infections caused by microorganisms that are resistant to antibiotics often lead to the longer hospital stays associated with higher medical costs and increased mortality [1]. There are different indications worldwide suggesting that the incidence of antibiotic resistance will further increase. One such indication is the COVID-19 outbreak, which is raising concerns regarding the excessive use of antimicrobials and biocides that could have an impact on AMR escalation [2]. The spread of infections caused by the antibiotic resistant bacteria is continuously reported worldwide. One piece of evidence for this claim can be observed in the region of the Nordic countries, where until 2000 very low numbers of MRSA cases were recorded. However, the numbers of MRSA cases increased 6–35 fold until 2016 [3]. Likewise, according to the European Centers for Disease Prevention and Control in 2015, 13.7% of *Pseudomonas aeruginosa* isolates were resistant to at least three classes of antimicrobials, while according to United States data, multidrug resistant (MDR) *P. aeruginosa* is the cause of 13% of severe healthcare-associated infections [4]. MDR strains of *Acinetobacter baumannii* found in hospitals can spread epidemically and are the reasons for increased patient mortality [5]. One of the factors enhancing both the persistence and resistance of these bacteria is their ability to establish biofilms, the virulence factor associated with the chronic nature of microbial infection [6]. Microbial biofilm is a complex community composed of microorganisms which are adhered to the biotic or abiotic surface areas and surrounded by an extracellular matrix containing extracellular DNA (eDNA), proteins, and polysaccharides [7]. Biofilms contribute to the antibiotic resistance due to their complex structure that interferes with antibiotic bioavailability and may reduce antibiotic levels within the biofilm though the lowering of its efficacy [8]. Components of the extracellular matrix such as the polysaccharides and eDNA contribute to the antibiotic resistance due to interference with antibiotic penetration and the enabling of the horizontal transfer of antibiotic resistance genes [9]. Combinatorial treatment as the strategy to eliminate biofilms by targeting both the extracellular polymeric matrix and the microbial cells has been suggested [10].

A recent review has provided insight into antivirulence as an alternative antimicrobial strategy that is based on the obstruction of bacterial virulence factors rather than on bacterial growth. Some of the drugs with an antivirulence approach have already been approved, while others are undergoing clinical studies. The advantage for using the antivirulence-based approach is the reduced selection pressure that would reduce the chances for resistance development, as well as the minimal perturbation of the healthy microbiota [11]. Among the wide palette of antivirulence strategies studied so far are the quenching of pathogen quorum sensing systems, the disassembling of bacterial functional membrane microdomains, as well as the interference with biofilm formation and bacterial toxins [12]. However, efficacy of an antivirulence agent can be interfered by the emergence of insensitive bacterial mutants, suggesting that more detailed research is needed for the development of antivirulence drugs that would not induce resistance [13].

Some of the studied antivirulence agents include 2-aminobenzothiazoles, compounds that reduce the expression of virulence genes in *Salmonella enterica* [14]. Other antivirulence targets are bacterial histidine kinases, parts of the two-component system that regulates virulence, and these have been targeted by thiophene derivatives [15] and a novel thienopyridine compound [16]. Likewise, quinazoline-based compounds target the PhoP/PhoQ signal transduction system, which is crucial for virulence in *Salmonella* [17], while the Hsp90 inhibitor, radicicol, interacts with the ATP-binding pocket of sensor kinase PhoQ and inhibits the autokinase activity of the PhoQ cytoplasmic region [18].

Polyphenols are a group of compounds mainly originating from plants and are among the most widely examined phytochemicals [19]. Polyphenols are well-known for their antioxidant, cardio-protective, anti-inflammatory, and antimicrobial bioactive properties [20,21]. These compounds are part of the regular human diet; it has been proven that polyphenol-rich diets could prevent most chronic diseases [22], with some authors claiming that the studies using isolated food polyphenols could not link its activity to epidemiological observations made for the whole foods [23]. Polyphenols have also been abundantly studied as antimicrobials, but mainly towards the antibiotic susceptible microorganisms [24].

Despite the availability of a range of antibacterial drugs on the current pharmaceutical market worldwide, we need to further study potential antimicrobial agents, preferably the ones employing novel antimicrobial mechanisms, in order to avoid cross-resistance with the current anti-bacterial palette and to provide efficient therapeutic strategies for the infections caused by bacteria that are resistant to the current antibiotic arsenal. Likewise, the biofilm’s structure is mostly resistant to the treatment with current antibacterial palette, making the development of efficient antibiofilm agents of high priority. The aim of this study was to determine the potential of twelve different polyphenols: hesperetin, hesperidin, naringenin, naringin, taxifolin, rutin, isoquercitrin, morin, chlorogenic acid, ferulic acid, *p*-coumaric acid, and gallic acid to inhibit the growth of eleven antibiotic resistant strains of bacteria. Likewise, we aimed to elucidate their antibiofilm potential and cytotoxicity to human keratinocytes. Based on the previous assays, we have selected one polyphenol (rutin) as the representative of the group to study its antibiofilm, antivirulence and antimicrobial mechanisms on the cellular, biochemical and molecular levels.

## 2. Results

### 2.1. Polyphenols Are Able to Inhibit Growth of Antibiotic Resistant Bacteria

The selected polyphenols were able to inhibit the growth of antibiotic resistant bacterial species, with activities listed as promising, such as in the case of morin, with the lowest MIC of 0.12 mg/mL towards *MRSA* IBRS MRSA 011 and *P. aeruginosa* IBRS P001. On the other hand, low activity was recorded for naringin and gallic acid, polyphenols that were inactive (MIC > 1 mg/mL) towards nine and seven examined bacterial strains, respectively (Table 1).

Hesperetin, compared to its glycoside, hesperidin, exhibited identical MICs towards majority of examined bacterial species. On the other hand, naringenin and its glycoside, naringin, have exhibited different inhibitory capacities towards examined microorganisms, with the antimicrobial potential of naringenin being more promising compared to naringin. Derivatives of quercetin, taxifolin, rutin, and isoquercitrin have shown promising antibacterial capacity, along with morin. Isoquercitrin and taxifolin have shown promising inhibitory potential, with the lowest MIC of 0.25 mg/mL. Among the examined phenolic acids, more promising activity could be observed for ferulic acid and *p*-coumaric acid compared to gallic and chlorogenic acid.

### 2.2. Polyphenols Reduce the Ability of P. aeruginosa IBRS P001 to Form Biofilms

**Figure 1:**
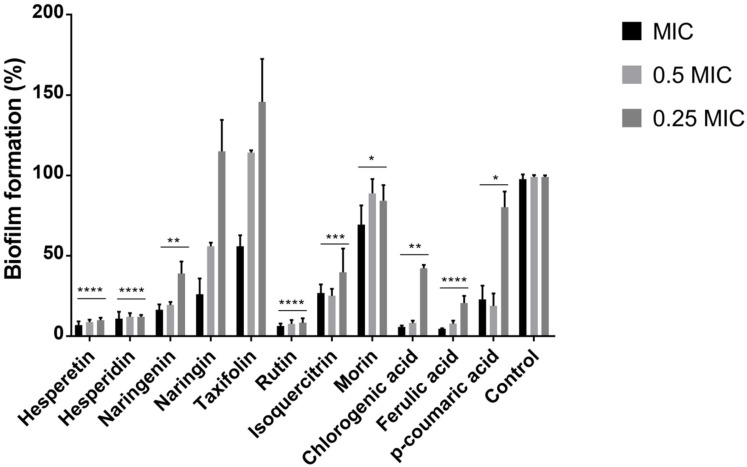
Formation of *P. aeruginosa* IBRS P001 biofilm after treatment with polyphenols. The error bars indicate standard deviations. The asterisks represent statistical significance *, *p* ≤ 0.05; **, *p* ≤ 0.01; ***, *p* ≤ 0.001, ****, *p* ≤ 0.0001, the data are presented as mean ± SD.

We have observed a significant (****, *p* < 0.0001) reduction in *P. aeruginosa* IBRS P001 biofilm formation upon treatment with hesperetin, hesperidin, rutin, and ferulic acid. A promising (***, *p* ≤ 0.001) potential to reduce the biomass of *P. aeruginosa* IBRS P001 biofilms was noticed for isoquercitrin. On the other hand, biofilm formation was not significantly altered upon treatment with naringin and taxifolin (Figure 1).

Both derivatives of quercetin that were studied, rutin and isoquercitrin, have shown significant antibiofilm capacity. Hesperetin and its glycoside, hesperidin, were both among the most active antibiofilm compounds. On the other hand, the flavanone naringenin has shown stronger antibiofilm potential compared to its glycoside naringin.

### 2.3. Polyphenols Are Mainly Non-Cytotoxic towards HaCaT Cells

The cytotoxicity of polyphenols towards HaCaT cells was examined (Table 2). Their non-cytotoxic nature could be observed for the majority of the examined compounds (IC_50_ > 1 mg/mL). On the other hand, the cytotoxic effect was recorded for gallic acid (IC_50_ < 0.08 mg/mL), chlorogenic acid (IC_50_ 0.279 mg/mL), morin (IC_50_ 0.347 mg/mL), and taxifolin (IC_50_ 0.495 mg/mL).

By comparing derivatives of different polyphenols, it was noticed that naringenin exhibits more profound cytotoxicity compared to its glycoside naringin, IC_50_ 0.528 mg/mL and >1 mg/mL, respectively; while hesperetin and its glycoside, hesperidin, have both shown non-cytotoxic properties. The cytotoxicity of aglycones (naringenin, taxifolin and morin) was more profound compared to the cytotoxicity displayed by glycosides.

### 2.4. Rutin Significantly Affected Urinary Catheter Biofilms

**Figure 2:**
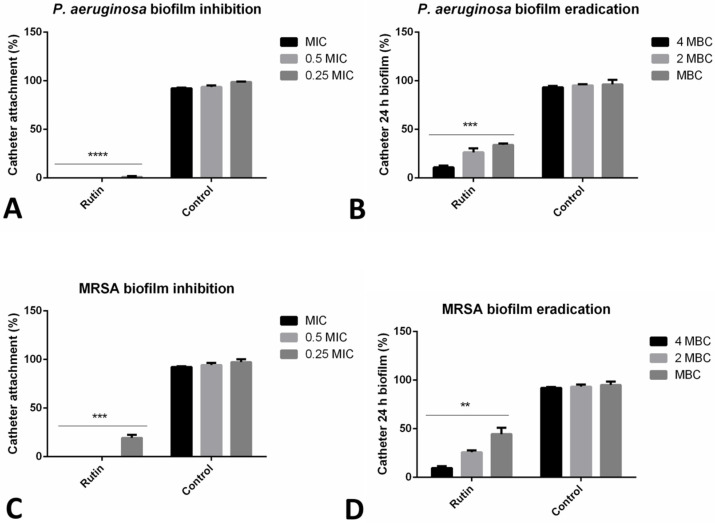
Impact of rutin on biofilm prevention and biofilm eradication on catheter biofilm model. *P. aeruginosa* IBRS P001 cell attachment on catheter surface (**A**); 24 h old *P. aeruginosa* biofilm (**B**); *MRSA* IBRS MRSA011 cell attachment on catheter surface (**C**) and 24 h old MRSA biofilms (**D**) upon treatment with rutin. The asterisks represent statistical significance **, *p* ≤ 0.01; ***, *p* ≤ 0.001; ****, *p* ≤ 0.0001, the data are presented as mean ± SD.

The inhibitory effect of rutin on the biofilms in the model of urinary catheter biofilm was examined. Incubation with rutin, even in the sub-MIC concentration has significantly reduced (***, *p* ≤ 0.001, ****, *p* ≤ 0.0001) the biofilm forming abilities of two strains examined (*MRSA* IBRS MRSA 011 and *P. aeruginosa* IBRS P001) (Figure 2). The number of *P. aeruginosa* IBRS P001 colonies attached to the urinary catheters after the treatment with MIC and 0.5 MIC of rutin was 0, indicating 100% biofilm inhibition as determined using this model.

*P. aeruginosa* IBRS P001 and *MRSA* IBRS MRSA 011 catheter biofilms 24 h old were successfully eradicated with rutin (10.9%, and 9.6% of biofilm cells remained viable after the treatment, respectively). The effect of rutin on the eradication of catheter biofilms was concentration-dependent. Rutin at 2 MBC eradicated *P. aeruginosa* IBRS P001 and *MRSA* IBRS MRSA 011 biofilms for 73.7% and 74.2%, respectively (Figure 2).

### 2.5. Co-Incubation with Rutin Reduced Bacterial Adhesion to HaCaT

**Figure 3:**
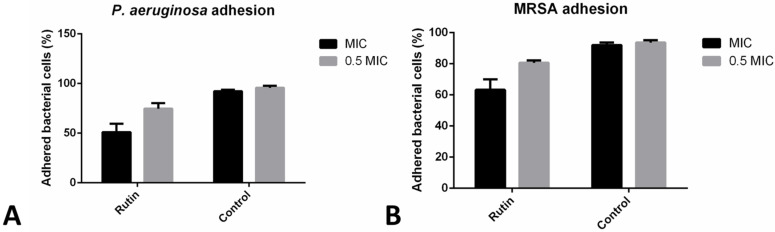
*P. aeruginosa* IBRS P001 (**A**) and *MRSA* IBRS MRSA011 (**B**) adhesion to HaCaT cells upon treatments with rutin (%). The data are presented as mean ± SD.

The incubation with rutin (MIC) has reduced the CFU of both examined strains, *P. aeruginosa* IBRS P001 and *MRSA* IBRS MRSA 011, which were able to adhere to the surface of human keratinocytes for 51% and 63%, respectively. A lower concentration (0.5 MIC, 0.25 mg/mL) reduced the number of adhered *P. aeruginosa* IBRS P001 CFUs for 25%; while its effect on *MRSA* IBRS MRSA 011 was lower (19% inhibition) (Figure 3).

### 2.6. Co-Incubation with Rutin Affects Different Aspects of Biofilm Formation

**Figure 4:**
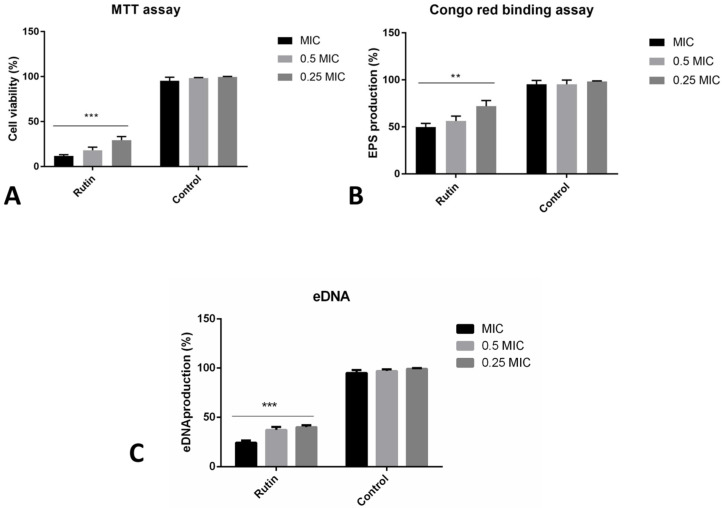
Different modes of rutin acting on *P. aeruginosa* IBRS P001 biofilm formation: impact on viability of cells in biofilm (**A**); production of exopolysaccharides (**B**) and eDNA production (**C**). The asterisks represent statistical significance **, *p* ≤ 0.01; ***, *p* ≤ 0.001, the data are presented as mean ± SD.

Co-incubation with rutin has led to the significant reduction of the formed biofilm biomass, as recorded in the previous crystal violet biofilm assay (Figure 1). We have additionally observed different rutin modes of *P. aeruginosa* IBRS P001 biofilm prevention abilities (Figure 4). The metabolic activity of cells forming the biofilm was reduced by 88% when bacteria were co-incubated with rutin MIC (Figure 4A). The level of biofilm exopolysaccharides was also significantly (**, *p* ≤ 0.01) affected with the application of rutin, as determined in the Congo red binding assay (Figure 4B). Rutin at MIC, 0.5 MIC and 0.25 MIC reduced the levels of the produced exopolysaccharides to 50%, 56% and 72% of production, respectively, compared to the vehicle treated control. Likewise, rutin’s co-incubation has induced a significant (***, *p* ≤ 0.001) reduction in the amounts of eDNA within the bacterial biofilm. Even the lowest examined concentration (0.25 MIC, 0.125 mg/mL) induced more than a 50% inhibition of eDNA levels (Figure 4C).

### 2.7. Rutin Can Eradicate Pre-Formed Biofilms by Diverse Mechanisms

**Figure 5:**
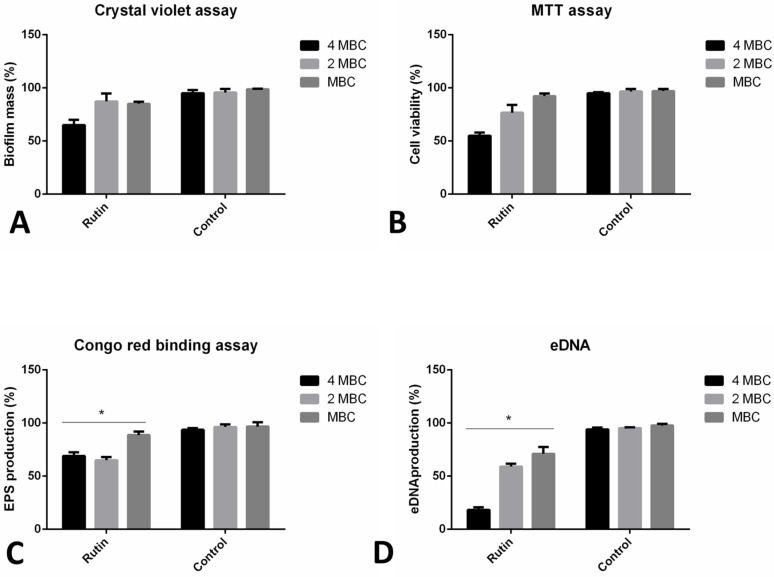
Different mechanisms of *P. aeruginosa* IBRS P001 24 h old biofilms eradication: impact on biofilm mass (**A**); interference with cell viability (**B**); exopolysaccharide (**C**) and eDNA (**D**) production. The asterisks represent statistical significance *, *p* ≤ 0.05; the data are presented as mean ± SD.

The impact of rutin was examined towards 24 h old established *P. aeruginosa* IBRS P001 biofilms by different assays (Figure 5). The crystal violet assay has indicated that after 24 h of rutin treatment (examined concentration equal to 4 MBC—4 mg/mL), *P. aeruginosa* biofilms were significantly reduced compared to the original biomass (35% inhibition) (Figure 5A). Lower concentrations of rutin have induced only a slight eradication of formed biofilm biomass (13% and 15% inhibition, respectively).

The viability of cells within the biofilms was altered upon the application of rutin (reduced to 55% viability at the maximal concentration examined), as indicated by assessing the bacterial metabolic activity (Figure 5B). The lowest tested concentration (MBC, 1 mg/mL) had almost no effect on the bacterial viability in the biofilms (92% of the cells remained viable).

Rutin treatment induced lower amounts of exopolysaccharide production within the biofilm, as determined in the Congo red binding assay (Figure 5C). Exopolysaccharide production decreased to 69%, 65%, and 89% after the application of rutin 4 MBC, 2 MBC, and MBC, respectively. Levels of eDNA in the biofilm matrix were significantly reduced upon rutin treatment (Figure 5D).

### 2.8. Rutin Suppresses P. aeruginosa IBRS P001 Virulence Factors Production

**Figure 6:**
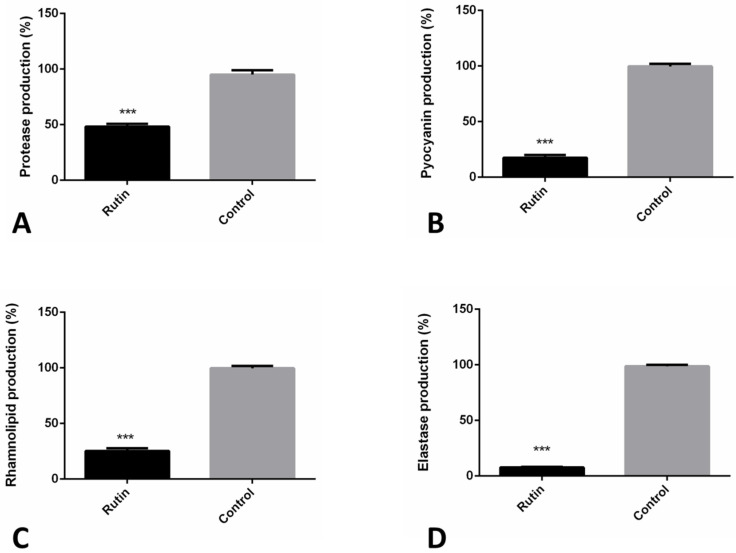
Rutin (0.5 MIC, 0.250 mg/mL) antivirulence mechanisms towards *P. aeruginosa* IBRS P001: impact on protease (**A**); pyocyanin (**B**); rhamnolipid (**C**) and elastase (**D**) production. The asterisks represent statistical significance ***, *p* ≤ 0.001; the data are presented as mean ± SD.

The impact of rutin on *P. aeruginosa* IBRS P001 model strain extracellular virulence factors production was examined (Figure 6). We have studied the production of the proteases, pyocyanin, elastase, and rhamnolipid in vitro using corresponding spectrophotometric assays. Rutin significantly reduced production (***, *p* ≤ 0.001) of *P. aeruginosa* IBRS P001 extracellular virulence factors; elastase production was reduced for more than 90% (91%), while production of pyocyanin and rhamnolipid was decreased for 82.1% and 74.4%, respectively, compared to the control. Protease production was decreased for more than 50%. These results demonstrate that rutin significantly suppresses the *P. aeruginosa* IBRS P001 extracellular virulence factor production when applied in a dose of 0.25 mg/mL (0.5 MIC).

### 2.9. Rutin Suppresses the P. aeruginosa IBRS P001 QS Systems Expression

**Figure 7:**
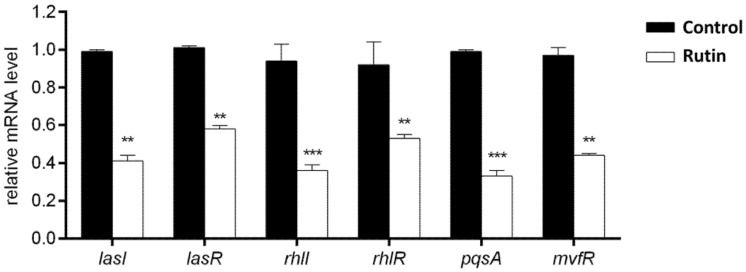
Relative mRNA levels of different *P. aeruginosa* IBRS P001 virulence associated genes upon treatment with rutin (0.5 MIC, 0.250 mg/mL). The asterisks represent statistical significance **, *p* ≤ 0.01; ***, *p* ≤ 0.001, the data are presented as mean ± SD.

RT-qPCR was performed in order to confirm the above described effects of rutin on *P. aeruginosa* IBRS P001 virulence (Figure 7). The selected *P. aeruginosa* genes belong to three QS networks las, rhl, and pqs: las-lasI, lasR; rhl-rhlI, rhlR; pqs-pqsA, mvfR. The acquired results revealed that the treatment of *P. aeruginosa* IBRS P001 with rutin (0.5 MIC, 0.25 mg/mL) significantly decreased the relative mRNA levels of analyzed QS genes (**, *p* ≤ 0.01; ***, *p* ≤ 0.001). The most significant downregulation was observed for the autoinducer synthases rhlI and pqsA (2.6 and 3-fold lower, respectively; ***, *p* ≤ 0.001) when compared to the untreated control. The relative expression of the other four analyzed genes was decreased: 2.4 times for lasI, 2.2 times for mvfR and about 1.7 times for lasR and rhlR.

### 2.10. Rutin’s Impact on Cell Membrane Permeability

**Figure 8:**
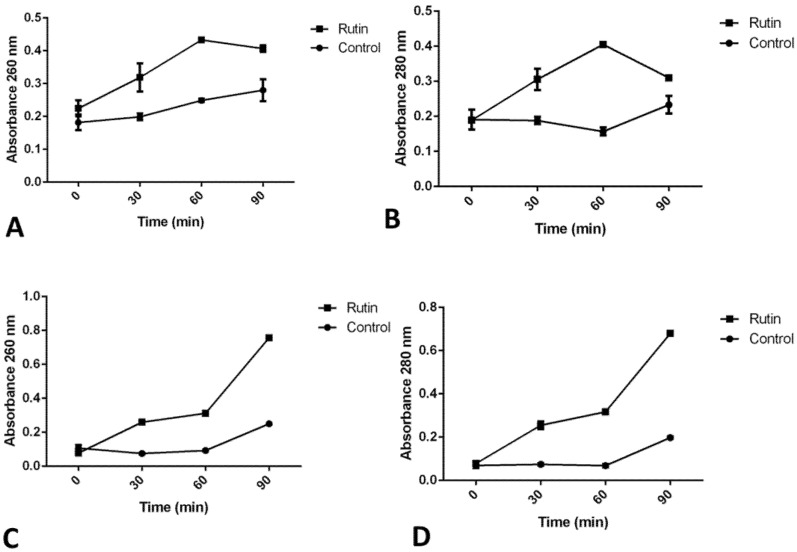
Leakage of intracellular material after treatment with rutin (MIC) as detected for *P. aeruginosa* IBRS P001 nucleic acids (**A**) and proteins (**B**), as well as for *MRSA* IBRS MRSA 011 nucleic acids (**C**) and proteins (**D**). The data are presented as mean ± SD.

Treatment with rutin has obstructed *P. aeruginosa* IBRS P001 cell membrane integrity, as indicated with the increase in absorbance of both released nucleic acids (Figure 8A) and proteins (Figure 8B) after 60 min. Membrane integrity of *MRSA* IBRS MRSA 011 was also disrupted, but in this case a more significant increase in the absorbance was observed after 90 min treatment, as indicated by the absorbance at 260 nm (Figure 8C) and 280 nm (Figure 8D).

## 3. Discussion

This is the first study that examined the antibacterial capacity of 12 different polyphenols towards 11 bacterial strains with diverse range of resistance to antimicrobial therapy. The majority of previous research was focused on the determination of the minimal inhibitory concentration of polyphenols towards the antibiotic susceptible strains [25,26]. Likewise, this is the first parallel study of antibiofilm capacity of 12 selected polyphenols on antibiotic resistant *P. aeruginosa* IBRS P001. Rutin, the polyphenol selected as the most promising based on the antimicrobial, antibiofilm and cytotoxic assay, displayed wide antibiofilm capacity towards *P. aeruginosa* and MRSA biofilms, which has not been examined previously in such detail. Our study further elucidated the antivirulence capacity of rutin along with its mechanisms, suggesting it as a promising antimicrobial agent employing a diverse mechanism of activities.

Hesperetin’s MIC determined previously was 1 mg/mL or higher [27] towards the strains of *Escherichia coli* K-12 MG1655, *Salmonella enterica* serovar Typhimurium LT2, and *Pseudomonas putida* ATCC 795. We have observed a similar antibacterial effect (MIC 0.5 mg/mL or higher) towards a range of antibiotic-resistant bacterial strains. Similarly, hesperidin exhibited MIC 0.5 mg/mL or higher (Table 1) as was also observed in the previous study (MICs 1.13, 1.53 and 1.27 mg/mL towards *E. coli* (0157:H7 ATCC 51659), *S. aureus* (ATCC 25923) and *P. aeruginosa* (NRRLB-272), respectively [28]. Different naringenin derivatives have been tested as growth inhibitors of antibiotic resistant bacterial strains, and MICs 4—1024 µg/mL towards methicillin resistant *S. aureus* were observed [29]. Since our study observed MIC towards MRSA equal to 0.250 mg/mL, this implies that certain structural modifications might lead to the higher anti-MRSA efficacy. Likewise, the range of MICs for naringenin derivatives towards beta-lactam-resistant *A. baumanii* were 512 µg/mL or higher [29] compared to MICs higher than the 1000 µg/mL observed in Table 1. The lowest MIC of naringin was towards *P. aeruginosa* IBRS P001 (0.25 mg/mL). Similar potency was observed previously and presented as IC_50_ 0.570 mg/mL against *P. aeruginosa* MTCC 2488 [30]. Bactericidal concentration (1.4 mg/mL) was in the range of MBCs observed in our study (0.5 mg/mL or higher than 1 mg/mL) towards *P. aeruginosa* strains [30]. Taxifolin displayed MIC 512 µg/mL towards *S. aureus* USA300, methicillin resistant bacterial strain [31], while MIC observed towards resistant *S. aureus* in our study (0.250 mg/mL) was more promising. Rutin previously displayed MIC at 800 µg/mL towards multiple drug resistant *P. aeruginosa* [32], similar to the our investigation (MIC 500 µg/mL). Contrary to the current study of isoquercitrin towards MRSA (MIC 0.5 mg/mL), a previous study has shown no inhibitory activity towards other *S. aureus* strains (*S. aureus* Newman and *S. aureus* Newman Δ*coa*) in concentrations up to 1024 µg/mL [33]. On the other hand, the research on *S. aureus* clinical isolate and *S. aureus* ATCC 11632 suggested its more promising antibacterial activity with MICs 0.2 mg/mL and 0.1 mg/mL, respectively [34]. The previous MIC recorded for morin was 32 µg/mL towards MRSA T144 [35], while we have highlighted it as the most active flavonoid towards MRSA, but with higher MIC compared to the previous research (125 µg/mL). Chlorogenic acid has previously been tested as a growth inhibitor of three *K. pneumoniae* strains (MICs 2047 and 2048 µg/mL) [36], while in this research it also exhibited a MIC higher than 1000 µg/mL towards two strains examined. Ferulic acid has been previously examined, and it displayed MICs of 100, 100 and 1100 µg/mL towards *E. coli* CECT434, *P. aeruginosa* ATCC 10145, and *S. aureus* CECT 976 [37], respectively. Likewise, its lowest MICs towards antibiotic resistant strains were 0.5 mg/mL. A previous investigation of *p*-coumaric acid described its efficacy in combating *E. coli* (fluoroquinolones and ampicillin-resistant) with MIC 1 mg/mL, which is identical, as here determined, towards *E. coli* IMD989. Gallic acid has previously shown MIC 0.5 mg/mL towards *E. coli* ATCC 25922 [38], while in our study it exhibited MIC higher than 1 mg/mL towards *E. coli* strains.

Polyphenols with the most promising antibiofilm potential at concentrations of 0.25 MIC-MIC were hesperetin, hesperidin, rutin, and ferulic acid. Sub-MIC doses of rutin were examined previously, with the highest inhibition at 400 µg/mL (45.33%) towards the resistant strain *P. aeruginosa* MTCC 2488 [32]. With a similar concentration (MIC, 500 µg/mL), only 6% of the biofilm biomass was formed after treatment, indicating 94% inhibition (Figure 1). The differences in the used strains or the methodology might be the reason for the discrepancies e.g., 48 h incubation [32] vs. 24 h biofilm incubation (our study). Ferulic acid was able to reduce *P. aeruginosa* ATCC 10145 biofilms for more than 70% with 1000 µg/mL [37], while in our study 500 µg/mL induced an even higher inhibition. For hesperetin and hesperidin, according to the author’s best knowledge, this is the first evidence of the antibiofilm effects on *P. aeruginosa* and according to the obtained results these two flavonoids should be further examined as promising antibiofilm agents.

The compounds hesperetin, hesperidin, naringin, rutin, isoquercitrin, ferulic, and *p*-coumaric acid displayed no cytotoxicity at maximal tested concentrations (1 mg/mL). On the other hand, gallic acid exhibited the lowest IC_50_ (<0.080 mg/mL) while the previous assessment established IC_50_ 84.2 µg/mL towards HaCaT cells [39]. Other researchers have used different normal human cells such as human lung embryonic fibroblasts (TIG-1) and human umbilical vein endothelial (HUVE) cells [40] and human normal liver cells L-02 [41].

Rutin at MIC and sub-MIC concentrations efficiently inhibited catheter *P. aeruginosa* and MRSA biofilms. Biofilms establishment on the urinary catheters leads to persistent and antibiotic-resistant infections [42]. Previous research on two polyphenols, quercetin and myricetin, proved their efficacy in reducing *S. aureus* catheter colonization [43]. However, according to the authors best knowledge, it was not yet examined whether rutin is able to prevent/eradicate *P. aeruginosa* and MRSA catheter biofilms.

The application of rutin has also reduced the ability of bacteria cells to adhere to human keratinocytes. It was previously shown that rutin interfered with the attachment of both A549 and HT29 cells to fibronectin and to collagen type I and IV [44], so it seems that rutin displays wide anti-adhesion capacity. As recently reviewed, anti-adhesion-based therapies can be useful in the prevention and treatment of bacterial infections [45].

Rutin has displayed a stronger effect in preventing *P. aeruginosa* biofilm formation compared to its eradication ability, even though higher concentrations of rutin were applied in eradication assays, which is in accordance with the expected higher resistance of mature biofilms [46].

Sub-MIC concentrations of rutin (50–400 µg/mL) were previously shown to induce only up to 50% inhibition, possibly due to the longer incubation-48 h [32]. Rutin (0.5 MIC, 0.250 mg/mL) notably reduced the levels of produced exopolysaccharides (44% inhibition). EPS production was also previously established to decrease upon application of rutin (more than 40% inhibition with 200 µg/mL of rutin) [32]. Other researchers have examined total flavonoids of 1600 µg/mL, from *Potentilla kleiniana*, with rutin as the dominant flavonoid, and found that they have inhibitory effects towards biofilm eDNA [47].

The antivirulence spectrum of rutin was studied at the dose of 0.5 MIC (0.250 mg/mL). Rutin was able to significantly (***, *p* ≤ 0.001) reduce the production of four different *P. aeruginosa* virulence factors: protease, pyocyanin, rhamnolipid, and elastase. According to the authors’ best knowledge, rutin’s antivirulence effect on *P. aeruginosa* has not been elucidated previously. This is the first study to investigate the molecular mechanisms behind this virulence inhibition. Flavonoids have been previously shown to specifically inhibit quorum sensing through the antagonism of the autoinducer-binding receptors *lasR* and *rhlR* [48], as shown also in our study of rutin. Previous studies have shown that rutin reduces the expression of quorum sensing genes in *E. coli* [49].

Rutin ’s treatment has led to the release of proteins and nucleic acids from *P. aeruginosa* and *MRSA* bacterial cells, suggesting membrane interference as one of the antimicrobial mechanisms that rutin employs. Previously, apigenin, apigetrin, and astragalin were shown to induce nucleic acid leakage [50,51]. The property of flavonoids to interact with membranes is found to correlate with their bioactivities [52]. Likewise, polyphenols such as gallic and ferulic acid induce an antimicrobial effect through irreversible changes in membrane properties such as charge, intra and extracellular permeability, and physicochemical properties. They induce local damages or pore formation in the cell membranes that subsequently lead to the leakage of intracellular material [53].

Besides the wide antivirulence potential observed for rutin in this study, a range of biological activities of this polyphenol were previously recorded. Among others, rutin displayed antidiabetic [54], anticancer [55], as well as neuroprotective activity [56].

The majority of examined polyphenols could reduce the planktonic and biofilm growth of antibiotic resistant bacteria in active concentrations that were non-cytotoxic to human cells. Rutin displayed wide antibiofilm capacity by employing diverse mechanisms that could be utilized for the further development of novel antimicrobials and novel antivirulence strategies.

## 4. Materials and Methods

### 4.1. Antimicrobial Assay

Resistant bacterial strains used in the antimicrobial assays are shown in Table 3. The bacterial suspension (0.5 Mc Farland) was prepared, and twenty-fold dilutions of these suspensions were mixed in a 1:10 ratio with the corresponding polyphenol dilution (0.0039–1 mg/mL). Microtiter plates were incubated at 37 °C for 24 h. The lowest concentrations without visible growth (under a binocular microscope) were defined as MICs. The bactericidal concentrations (MBCs) were determined by serial subcultivation of 2 μL of the well’s content and further incubation for 24 h at 37 °C. Streptomycin (SigmaAldrich, Darmstadt, Germany) was used as a positive control. The polyphenols were obtained from Extrasynthese, Rhone, France (rutin, isoquercitrin) and SigmaAldrich, Germany (hesperetin, hesperidin, naringenin, naringin, taxifolin, morin, chlorogenic acid, ferulic acid, *p*-coumaric acid, and gallic acid). The stock compounds were prepared in ethanol (30%), and adequate ethanol concentrations were used as the negative control in the assays. Experiments were done in triplicate.

### 4.2. Crystal Violet Antibiofilm Assay

*P. aeruginosa* IBRS P001 was incubated with MIC and sub-MIC of the tested compounds in tryptic soy broth enriched with glucose (2%) at 37 °C for 24 h, as described earlier [66] with some modifications. After incubation, each well was washed twice with sterile PBS (phosphate-buffered saline, pH 7.4) and fixed with methanol for 10 min. After removal of methanol the plate was air-dried. The biofilm was stained with 0.1% crystal violet (Bio-Merieux, Marcy l’Etoile, France) for 30 min. The plate was washed with water, air dried, and the stain was dissolved with 200 μL of 96% ethanol (Zorka, Sabac, Serbia). The absorbance was read at 620 nm on a Multiskan™ FC microplate photometer (Thermo Scientific™, Waltham, MA, USA). The results are presented as biofilm forming capacity (%) compared to control.

### 4.3. Evaluation of Cytotoxicity in HaCaT Cell Line

For determination of the polyphenols’ cytotoxic effect, a crystal violet assay was used [67], with modifications. The cytotoxic effect of polyphenols was analyzed on spontaneously immortalized human skin keratinocytes (HaCaT, AddexBio No. T0020001, San Diego, CA, USA) cell lines. Cells were grown in a high-glucose Dulbecco’s Modified Eagle Medium (DMEM, Gibco, Waltham, MA, USA) supplemented with 10% fetal bovine serum (FBS, Gibco, Waltham, MA, USA), 2 mM L-glutamine and 1% penicillin and streptomycin (Invitrogen, Waltham, MA, USA) at 37 °C in 5% CO_2_. Cells (1 × 10^4^ cells/well) were seeded in a 96-well plate (Sarstedt, Nümbrecht, Germany) 24 h before treatment. After the medium was aspirated, fresh medium supplemented with different concentrations of the polyphenols (0.0039–1 mg/mL) dissolved in PBS was added to the cells. Cells were incubated with the compounds for 24 h. The medium was removed and the cells were washed twice with PBS and stained for 15 min at room temperature with 0.5% crystal violet. After crystal violet was removed, the plates were washed in a stream of tap water and left to air-dry at room temperature for 24 h. The absorbance was measured in a microplate reader (Multiskan™ FC Microplate Photometer, Thermo Scientific™) at 590 nm. The results were expressed as IC_50_ value in μg/mL. The criterion used to categorize the cytotoxic activity polyphenols was as follows: IC_50_ ≤ 20 µg/mL = highly cytotoxic, IC_50_ ranged between 21 and 250 µg/mL = moderately cytotoxic, IC_50_ ranged between 251 and 500 µg/mL = weakly cytotoxic, and IC_50_ > 501 µg/mL = no cytotoxicity. All analyses were performed in triplicate; each replicate was also quantified three times. Data were expressed as mean standard deviation, where applicable.

For the following experiments, rutin was selected as the polyphenol with the most promising antimicrobial and antibiofilm activity, along with lack of cytotoxicity, based on the previous results.

### 4.4. Catheter Biofilm Inhibition/Eradication Model

The method was performed as described previously [68], with some modifications. Sterile silicon 16-mm Romed catheters (Van Oostveen Medical, Wilnis, The Netherlands) were cut into 1 cm lengths and placed in 24-well plates (Sarstedt, Nümbrecht, Germany). *P. aeruginosa* IBRS P001 and MRSA in TSB with 2% glucose (Torlak, Belgrade, Serbia) and rutin (0.25–1 MIC) were added for the inhibition assay. After incubation (37 °C, 24 h), the catheters were washed with PBS and transferred to 1.5 mL tubes. PBS was added and tubes were vortexed vigorously. The samples were diluted and seeded onto Plate Count Agar (HiMedia Laboratories, Mumbai, India). After being incubated at 37 °C for 24 h, the CFU (colony forming units) count was determined and the inhibition percentage relative to the untreated control was calculated.

For the catheter biofilm eradication assay, *P. aeruginosa* and MRSA were incubated in TSB with 2% glucose with 1 cm long catheters in 24-well plates at 37 °C for 24 h. After incubation, rutin (minimal bactericidal concentration, MBC-4MBC) was added to the plates and incubation continued for another 24 h. Subsequently, the catheters were washed and treated, as described above.

### 4.5. P. aeruginosa and MRSA Adhesion to HaCaT Cells

HaCaT cells were grown until confluence in 24-well plates with an adhesive bottom. The medium was removed and DMEM without FBS containing the rutin (MIC and 0.5 MIC) was added. The samples were incubated at 37 °C for 15 min, and bacteria (*P. aeruginosa* IBRS P001 and *MRSA* IBRS MRSA 011) were added. After incubation at 37 °C for 1 h, the cells were washed three times with DMEM without FBS and lyzed with 1 mL of 1% (*v*/*v*) Tween-20 (SigmaAldrich, Germany) at 37 °C for 30 min. Afterwards, dilutions of the bacterial suspension in each well were made and seeded on Plate Count Agar plates. The number of CFU was determined after incubation at 37 °C for 18 h, and the percentage of adhered bacterial cells was calculated following the equation:

% (adhesion relative to the control) = 100 × CFU (treated)/CFU (control)

With CFU (treated) representing the number of CFUs after the treatment of cells with rutin and CFU (control) representing the number of bacterial CFUs in the untreated sample. The solvent treated sample was used as the control.

### 4.6. Mechanisms of Rutin Biofilm Inhibition/Eradication

#### 4.6.1. Biofilm MTT Assay

The impact of rutin on *P. aeruginosa* IBRS P001 biofilm formation was determined as previously described [30] by estimating the metabolic activity of microbial cells using an MTT assay. Biofilms were allowed to develop at 37 °C in the presence of rutin in concentrations of 0.25–1 MIC for 24 h. The supernatants were then ejected and the biofilms washed with PBS. MTT reagent (200 μL, 0.5 mg/mL) was added and the plate was incubated at 37 °C in the dark for 2 h. The resulting dye was dissolved in DMSO and the absorbance was measured at 570 nm in a microtiter plate reader Multiskan™ FC Microplate Photometer, Thermo Scientific™. The inhibition percentage of biofilm cell viability was calculated:

%Inhibition = [(A570_control_ − A570_sample_)/A570_control_] × 100

With A570_control_ representing the absorbance of untreated sample, and A570_sample_ representing the absorbance of the rutin treated sample. A solvent-treated sample was used as the control.

#### 4.6.2. Congo Red Binding Assay

The estimation of rutin’s impact on biofilm exopolysaccharide production (EPS) was determined by the Congo red binding assay. *P. aeruginosa* IBRS P001 biofilm EPS production was determined as previously described [25]. Bacterial biofilms were formed in the presence of rutin (0.25–1 MIC) at 37 °C for 24 h. The planktonic cells were removed, and the adherent cells were washed with PBS. Wells were stained with 1% (*w*/*v*) Congo red (SigmaAldrich, Germany) in the dark for 30 min. Wells were aspirated and subsequently the bound dye was solubilized with 200 μL DMSO. Absorbance at 490 nm was measured in a microtiter plate reader Multiskan™ FC microplate photometer (Thermo Scientific™, United States) and the inhibition percentage of EPS production was calculated according to the following equation:

%Inhibition = [(A490_control_ − A490_sample_)/A490_control_] × 100

With A490_control_ representing the absorbance of the untreated biofilm and A490_sample_ the absorbance of the rutin treated sample. The solvent treated sample was used as the control.

#### 4.6.3. Quantification of eDNA

The quantity of eDNA was determined as previously described [69], with some modifications. *P. aeruginosa* IBRS P001 was grown in 96-well plates (Sarstedt, Germany) containing TSB enriched with 2% glucose and rutin (0.25–1 MIC), at 37 °C for 24 h. Subsequently, the planktonic cells were removed, and the wells were washed with PBS. TE buffer (Tris-EDTA buffer solution) was added to the wells and content was mixed vigorously by pipetting. The samples were transferred to 1.5 mL tubes and centrifuged at 10,000× *g* for 10 min. After removal of the supernatant, the pellet was suspended in a TE buffer by vortexing. Absorbance of the supernatant (260 nm) was measured after centrifugation at 10,000× *g* for 15 min. We calculated the inhibition percentage of eDNA relative to an untreated control. The solvent treated sample was used as the control.

#### 4.6.4. Rutin Biofilm Eradication Mechanisms

The bacterial cells were incubated in TSB enriched with 2% glucose in 96-well plates (Sarstedt, Germany) with adhesive bottom at 37 °C for 24 h. After incubation, wells were washed with PBS, and rutin (MBC–4 MBC) in TSB with 2% glucose was added. Incubation continued at 37 °C for another 24 h. The planktonic cells were removed.

The assays continued as described above for the crystal violet antibiofilm assay for the measurement of biofilm mass, biofilm MTT assay for the interference with cell viability, Congo red binding assay for estimation of exopolysaccharide production, and eDNA assay.

### 4.7. Extracellular Virulence Factors Analysis

#### 4.7.1. Protease Assay

The impact of rutin on the proteolytic activity of *P. aeruginosa* IBRS P001 was measured using azocasein as a substrate. Briefly, bacteria were grown in TSB broth in the presence and absence of rutin at 0.5 MIC concentrations at 37 °C for 16 h. Samples were centrifuged at 9660 g, room temperature, for 10 min (Heraeus biofuge stratos centrifuge, Thermo Electron Corporation, Waltham, MA, USA). Afterwards, the supernatant was mixed with azocasein (2%, SigmaAldrich, Germany) and incubated at 37 °C for 45 min. The reaction was stopped using 10% trichloroacetic acid and centrifuged at 9660× *g*, RT for 10 min. After centrifugation, the absorbance of the supernatants was measured at 440 nm and protease production in the treated sample calculated compared to the control (100% production).

#### 4.7.2. Elastase Assay

Culture supernatants of *P. aeruginosa* IBRS P001 previously incubated with 0.5 MIC (0.250 mg/mL) of rutin (10 h, 37 °C, aerobically) were mixed with Elastin-Congo red (Sigma-Aldrich, St. Louis, MI, USA) at a final concentration of 2 mg/mL [70]. After 48 h of incubation (37 °C, 180 rpm), the mixtures were collected and centrifuged at 15,700× *g* for 15 min. Quantification of the elastase activity was measured at 495 nm using a Plate Reader Infinite 200 pro (MTX Lab Systems, Vienna, VA, USA). An untreated culture supernatant was used as a positive control (supplemented with the solvent). The experiment was done in triplicate.

#### 4.7.3. Pyocyanin Assay

*P*. *aeruginosa* IBRS P001 was supplemented with rutin (0.5 MIC, 0.250 mg/mL) and cultivated for 10 h, at 37 °C, 180 rpm. Pyocyanin extraction from the culture supernatant was performed using chloroform in a 1:2 ratio [71]. The second extraction of the chloroform phase was done by 0.2 N HCl (3:1 ratio). The pyocyanin concentration was evaluated by recording the absorbance at 520 nm using a Plate Reader Infinite 200 pro (MTX Lab Systems, Vienna, VA, USA). The pyocyanin concentration was calculated by multiplication of the absorbance (A_520_) by 17.072. A solvent-treated culture supernatant was used as a positive control.

#### 4.7.4. Rhamnolipid Assay

Culture supernatants of the previously cultivated *P. aeruginosa* IBRS P001 strain incubated with rutin 0.250 mg/mL were acidified by using HCl to pH 2.0 [72]. Rhamnolipid production was measured spectrophotometrically at 570 nm by a Plate Reader Infinite 200 pro (MTX Lab Systems, Vienna, VA, USA). A solvent treated culture supernatant was used as a positive control.

### 4.8. RT-qPCR Analysis

Reverse transcription-quantitative PCR (RT-qPCR) was performed to reveal the impact of rutin on the *P*. *aeruginosa* IBRS P001 quorum sensing (QS) regulatory genes expression. Primers used for the RT-qPCR analysis are listed in Table 4. The total RNA was extracted from the *P*. *aeruginosa* IBRS P001 test strain (grown for 10 h, 37 °C, aerobically) cultivated in Mueller-Hinton medium and supplemented with rutin (0.250 mg/mL, final concentration) or without it by an RNeasy Mini Kit (Qiagen, Hilden, Germany). The same volume of the ethanol (solvent) was added in the positive control. The total RNA was then treated with DNase I using an Ambion DNA-freeTM Kit (ThermoFisher, Waltham, MA, USA) and reverse transcribed by a Rever-tAid RT Reverse transcription Kit (ThermoFisher, Waltham, MA, USA) according to the manufacturer’s instructions. Further amplification was achieved with FastGene IC Green 2× qPCR Universal Mix (Nippon Genetics, Dueren, Germany) in a 7500 Real-Time PCR System (Applied Biosystems, Waltham, MA, USA). Obtained data were normalized against the ribosomal gene *rpsL* as an internal control following the 2^−ΔΔCT^ method [73]. Experiments were done in triplicate.

### 4.9. Membrane Permeability Assay

The effect of rutin on membrane permeability (nucleotide and protein leakage) was evaluated as described earlier with some modifications [76]. The culture of *P. aeruginosa* IBRS P001 and *MRSA* IBRS MRSA 011 was incubated overnight at 37 °C, washed, and suspended in 10 mM PBS in order to achieve the final density of 10^8^ CFU/mL. Bacteria were incubated with rutin at a concentration equal to MIC for time intervals 0, 30, 60, and 90 min. Bacteria incubated with PBS were used as control. After incubation the mixture was filtered through a 0.22 μm pore size filter to remove the yeast cells. The optical density of the filtrate was measured at 260 and 280 nm with an Agilent/HP 8453 UV-Visible Spectrophotometer (Agilent Technologies, Santa Clara, CA, USA) at room temperature (25 °C).

### 4.10. Statistical Analysis

The experiments were performed in three replicates. The data were calculated as a mean ± standard error, and statistically analyzed using GraphPad PRISM 6 software by Student’s *t*-test, with *, *p* ≤ 0.05; **, *p* ≤ 0.01; ***, *p* ≤ 0.001; ****, *p* ≤ 0.0001.

## 5. Conclusions

The potential of polyphenols to combat the growth of antibiotic resistant bacteria in both planktonic and biofilm forms was confirmed in this study as well as the non-cytotoxic nature for the majority of the plant bioactive molecules examined. Rutin, as the most active representative, was proven to strongly affect the formation of bacterial biofilms in vitro as confirmed by a range of biofilm models and assays used. Likewise, rutin was able to reduce the production of *P. aeruginosa* virulence factors and to downregulate the expression of quorum sensing related genes of this pathogen. Its antimicrobial mechanism might be related to interference with membrane permeability. Rutin should be further studied and could be used for the development of novel antimicrobial therapeutics that might be efficient in combating antibiotic-resistant bacteria.

## Figures and Tables

**Table 1:** Antibacterial potential of selected polyphenols; results are expressed in mg/mL.

Bacteria		Hesperetin	Hesperidin	Naringenin	Naringin	Taxifolin	Rutin	Isoquercitrin	Morin	Chlorogenic Acid	Ferrulic Acid	*p*-Coumaric Acid	Gallic Acid	Streptomycin
*Methicillin resistant S. aureus* IBRS MRSA 011	MIC	0.5	0.5	0.25	0.5	0.25	0.5	0.5	0.12	0.5	0.5	0.5	>1	0.1
	MBC	1	1	0.5	1	0.5	1	1	0.25	1	1	1	>1	0.8
*P. aeruginosa* IBRS P001	MIC	0.5	0.5	0.25	0.25	0.25	0.5	0.25	0.12	0.5	0.5	0.5	>1	0.05
	MBC	1	1	0.5	0.5	0.5	1	0.5	0.25	1	1	1	>1	0.1
*P. aeruginosa* MMA83	MIC	1	1	1	>1	1	0.5	0.25	1	1	1	0.5	1	>1
	MBC	1	1	1	>1	>1	0.5	0.25	1	>1	1	1	1	>1
*E. coli* IBRS E003	MIC	>1	>1	0.5	>1	0.5	>1	>1	0.25	>1	>1	>1	>1	0.1
	MBC	>1	>1	1	>1	1	>1	>1	0.5	>1	>1	>1	>1	0.2
*E. coli* IMD989	MIC	>1	>1	1	>1	1	1	1	>1	>1	1	1	>1	1
	MBC	>1	>1	>1	>1	1	1	1	>1	>1	1	1	>1	>1
*A. baumannii* 6077/12	MIC	0.5	0.5	0.5	>1	0.5	0.5	0.25	0.5	1	0.5	0.5	0.5	>1
	MBC	0.5	0.5	0.5	>1	1	0.5	0.5	0.5	1	0.5	0.5	1	>1
*A. baumannii* S2/2	MIC	0.5	0.5	0.5	>1	0.5	0.5	0.5	0.5	1	0.5	0.5	0.5	>1
	MBC	0.5	0.5	0.5	>1	0.5	0.5	0.5	0.5	>1	0.5	0.5	0.5	>1
*K. pneumoniae* Ni9	MIC	1	1	0.25	>1	1	1	0.5	0.5	>1	1	1	1	>1
	MBC	1	1	0.5	>1	1	1	1	1	>1	1	1	1	>1
*K. pneumoniae* IT977	MIC	>1	1	1	>1	>1	1	1	>1	>1	1	1	1	>1
	MBC	>1	1	1	>1	>1	1	1	>1	>1	1	1	1	>1
*A. xylosoxidans* 37F	MIC	1	1	1	>1	1	0.5	0.5	0.5	1	1	1	0.5	>1
	MBC	1	1	1	>1	1	1	0.5	1	>1	1	1	1	>1
*S.* infantis 1371/1	MIC	>1	>1	>1	>1	>1	1	1	>1	>1	1	1	>1	>1
	MBC	>1	>1	>1	>1	>1	1	1	>1	>1	1	1	>1	>1

**Table 2:** Cytotoxicity of examined compounds towards HaCaT cell line, represented as IC_50_ (compound concentration that inhibited 50% of the cell growth) in mg/mL.

Polyphenol	IC_50_ (mg/mL)
Hesperetin	>1
Hesperidin	>1
Naringenin	0.528 ± 0.047
Naringin	>1
Taxifolin	0.495 ± 0.047
Rutin	>1
Isoquercitrin	>1
Morin	0.347 ± 0.016
Chlorogenic acid	0.279 ± 0.008
Ferulic acid	>1
p-coumaric acid	>1
Gallic acid	<0.080

**Table 3:** List of bacterial strains used in the study and the corresponding antibiotic resistance.

Strain	Resistance	Reference
Methicillin-resistant *Staphylococcus aureus* IBRS MRSA 011	cefoxitin	[57]
*Pseudomonas aeruginosa* IBRS P001	penicillin, ampicillin, amoxicillin, tetracycline, neomycin, gentamicin, ceftriaxone	[57]
*P. aeruginosa* MMA83	imipenem, meropenem, gentamycin	[58]
*Escherichia coli* IBRS E003	penicillin, ampicillin, amoxicillin, tetracycline, neomycin, gentamicin, ceftriaxone	[57]
*E. coli* IMD989	amoxicillin/clavulanate, ampicillin/sulbactam, piperacillin/tazobactam, cefuroxime, cefotaxime, ceftazidime, cefepime, imipenem, meropenem, trimethoprim/sulfamethoxazole, amikacin, gentamicin	[59]
*Acinetobacter baumannii* 6077/12	amoxicillin/clavulanate, ampicillin/sulbactam, piperacillin/tazobactam, cefoxitin, cefotaxime, ceftazidime, cefepime, aztreonam, imipenem, meropenem	[60]
*A. baumannii* S2/2	imipenem, meropenem, ciprofloxacin, levofloxacin, amikacin, gentamicin, tobramycin, trimethoprim/sulfamethoxazole, colistin	[61]
*Klebsiella pneumoniae* Ni9	imipenem, meropenem, colistin	[62]
*K. pneumoniae* IT977	ampicillin/clavulanate, piperacillin/tazobactam, cefazolin, ceftriaxone, cefepime, aztreonam, ertapenem, imipenem, meropenem, ciprofloxacin, moxifloxacin, gentamicin, tobramycin, nitrofurantoin	[63]
*Achromobacter xylosoxidans* 37F	tetracycline, chloramphenicol, ciprofloxacin, levofloxacin, trimethoprim/sulfamethoxazole	[64]
*Salmonella* Infantis 1371/1	tetracycline, quinolones, colistin	[65]

**Table 4:** Primers used for RT-qPCR analysis.

Gene	Primer Direction	Sequence (5′-3′)	Amplicon Size (bp)	Reference
*lasI*	Forward	GCGTGCTCAAGTGTTCAAGG	125	[74]
	Reverse	GGGCTTCAGGAGTATCTTCCTGG		
*lasR*	Forward	CTGTGGATGCTCAAGGACTAC	133	[75]
	Reverse	AACTGGTCTTGCCGATGG		
*rhlI*	Forward	CCATCCGCAAACCCGCTACATC	151	[74]
	Reverse	CTCCCAGACCGACGGATCGCTCGGC		
*rhlR*	Forward	GGGCGTGTTCGCCGTCCTGG	143	[74]
	Reverse	GGTATCGCTCCAGCCAGGCCTTG		
*pqsA*	Forward	GACCGGCTGTATTCGATTC	74	[75]
	Reverse	GCTGAACCAGGGAAAGAAC		
*mvfR*	Forward	GTCGGGACGGCTACAAGGTCG	129	[74]
	Reverse	GATTGCGCGGACCCTTGTTGAG		
*rpsL*	Forward	GCAACTATCAACCAGCTGGTG	231	[74]
	Reverse	GCTGTGCTCTTGCAGGTTGTG		

## Data Availability

Data is contained within the article.
